# Speckle tracking echocardiography and β-thalassemia major. A systematic review

**DOI:** 10.1007/s00277-023-05380-6

**Published:** 2023-08-01

**Authors:** Dimitrios Patsourakos, Constantina Aggeli, Yannis Dimitroglou, Sophia Delicou, Katerina Xydaki, Markos Koukos, Dimitrios Tsartsalis, Foteini Gialeli, Konstantinos A. Gatzoulis, Dimitrios Tousoulis, Konstantinos Tsioufis

**Affiliations:** 1 First Department of Cardiology, General Hospital of Athens Ippokrateio, National and Kapodistrian University of Athens, 114 Vasilissis Sofias Avenue, 11527 Athens, Greece; 2 Thalassemia and Sickle Cell Unit, General Hospital of Athens Ippokrateio, 114 Vasilissis Sofias Avenue, 11527 Athens, Greece

**Keywords:** Beta thalassemia major, Speckle tracking echocardiography, Myocardial deformation, Myocardial strain, Myocardial iron overload

## Abstract

Heart disease is among the primary causes of morbidity and mortality in β-thalassemia major (β-TM). Conventional echocardiography has failed to identify myocardial dysfunction at an early stage among these patients, thus speckle tracking echocardiography (STE) has been lately used. The objectives of this review were to 1) identify all published studies having evaluated myocardial strain among β-TM patients, 2) gather their results, 3) compare their findings and 4) propose recommendations based on these data. Literature search was conducted in PubMed, SCOPUS and Cohrane Library. Data regarding left ventricular global longitudinal (LV-GLS), circumferential (LV-GCS) and radial strain (LV-GRS), right ventricular longitudinal strain (RV-GLS), left and right atrial strain were extracted. Thirty-five studies (34 original articles and 1 meta-analysis) have met the inclusion criteria. LV-GLS has been reported being worse in patients compared to controls in 13 of 21 studies, LV-GCS in 7 of 11 studies, LV-GRS in 6 of 7 studies, RV-GLS in 2 of 3 studies and left atrial strain in all case–control studies. Myocardial iron overload (MIO) patient subgroups had worse LV-GLS in 6 of 15 studies, LV-GCS in 2 of 7 studies and LV-GRS in none of 7 studies. A small number of studies suggest left atrial strain correlation with electrical atrial ectopy and atrial fibrillation. It is suggested that STE should be applied supplementary to conventional echocardiography for early identification of myocardial dysfunction among β-TM patients. Potential myocardial strain utilities could be screening for myocardial iron overload, left ventricular diastolic dysfunction and atrial fibrillation.

## Introduction

Beta thalassemia major (β-TM) is a hereditary hemoglobinopathy characterized by total impairment of hemoglobin β-globin chain formation [1]. The consequent ineffective erythropoiesis and severe chronic hemolytic anemia renders β-TM patients transfusion dependent lifelong [2]. Regular blood transfusion therapy is initiated from early childhood and gradually will result in iron accumulation in vital organs (heart, liver and endocrine glands), unless appropriate iron chelation therapy is applied. Consequently, iron toxicity would result in myocardial, liver and endocrine disease [3]. In particular, heart disease is still the leading cause of death among β-TM patients, although the cardiac related deaths have decreased in absolute numbers in the recent years, reflecting the significant progress in iron chelation therapy [4]. Cardiac magnetic resonance (CMR) T2* imaging is currently considered the gold standard for detecting myocardial iron overload [5]. Iron chelation therapy tailored to CMR T2* has increased life expectancy of β-ΤΜ patients [6].

Transthoracic echocardiography is the most widely used modality to evaluate β-TM patients [7]. Nevertheless, conventional echocardiographic techniques have failed to recognize early myocardial dysfunction among these patients [8]. Chronic anemia results in high-output state with altered hemodynamic profile that renders the range of normal values of echocardiographic indices unsuitable for this particular patient population. In the last decade, speckle tracking echocardiography (STE), a novel echocardiographic technique, has been assessed in several specific patient populations to detect even subtle myocardial abnormalities [9], such as ischemic heart disease [10], valvular heart disease [11] and more importantly oncological patients having received chemotherapy [12]. Regarding β-TM, there are few data on the use of STE to overcome the limitations of conventional echocardiography.

The aim of the present review study is to search the literature for studies implementing STE in β-TM patients, gather and present the reported data, propose recommendations based on the findings and suggest possible design characteristics for future studies on this particular field.

## Materials and methods

### Protocol

The present study complies with the principles of the Preferred Reporting Items for Systematic Reviews and Meta-Analyses (PRISMA) guidelines [13].

### Eligibility criteria

Articles to be considered eligible for this systematic review should have met the following criteria:

i β-TM patients enrollment
ii Application of speckle tracking echocardiography to evaluate deformation of the cardiac chambers. Studies that evaluated segmental, but not global ventricular strain were not included.
iii Original articles regarding observational or experimental studies, including cross-sectional, case–control and cohort studies. Abstracts without corresponding manuscript and case-reports were not included.
iv Articles written in English.
v Articles published between 01–01-1990 and 31–08-2022.

### Sources and search strategy

The article search was performed on the following electronic databases: PubMed Database (https://pubmed.ncbi.nlm.nih.gov/), SCOPUS Database (https://www.scopus.com/search/form.uri?display=basic#basic) and Cochrane Library (https://www.cochranelibrary.com/search). The main key words used were “beta thalassemia major” that were combined with “speckle”, “deformation” and “strain” key words sequentially. To prevent missing articles, further checking was conducted manually in the reference lists of the emerged articles. The primary search was performed on 15th of February 2022, followed by an update search on 5th of September 2022.

### Article selection

All titles resulted from the aforementioned search process were reviewed in a preliminary screening by two independent reviewers (D.P, Y.D.) to identify articles meeting the inclusion criteria. A second level of screening was performed on the abstracts of the articles that qualified. Articles with unclear eligibility were reviewed in full text, while further checking was conducted manually in the reference lists of the emerged articles to identify missing studies. After applying the inclusion criteria, the final inclusion list was formed.

### Data collection

All articles from the final eligibility list were reviewed in full text and the following data were extracted and recorded in a custom worksheet that included the following variables: (i) author/year/country, (ii) type of study, (iii) number and mean age of subjects, (iv) male sex prevalence, (v) echocardiographic system being used, (vi) echocardiographic examination timing, (vii) magnetic scanner being used, (viii) CMR imaging timing, (ix) the particular deformation indices that were evaluated and the corresponding methods that were used, (x) CMR parameters, (xi) study exclusion criteria, (xii) percentage of patients with CMR evidence of MIO and mean T2* value, (xiii) strain values (LV GLS, GCS, GRS, RV GLS, LASr, LAScd, LASct, RASr) in each subject group separately, (xiv) strain values in MIO and non-MIO patient group respectively, (xv) hemoglobin concentration, (xvi) reported T2* correlation with any deformation index, (xvii) cut-off value of GLS predicting MIO. The corresponding authors of the selected studies were contacted via e-mail and requests for providing missing data were sent.

## Results

### Study selection

**Fig. 1:**
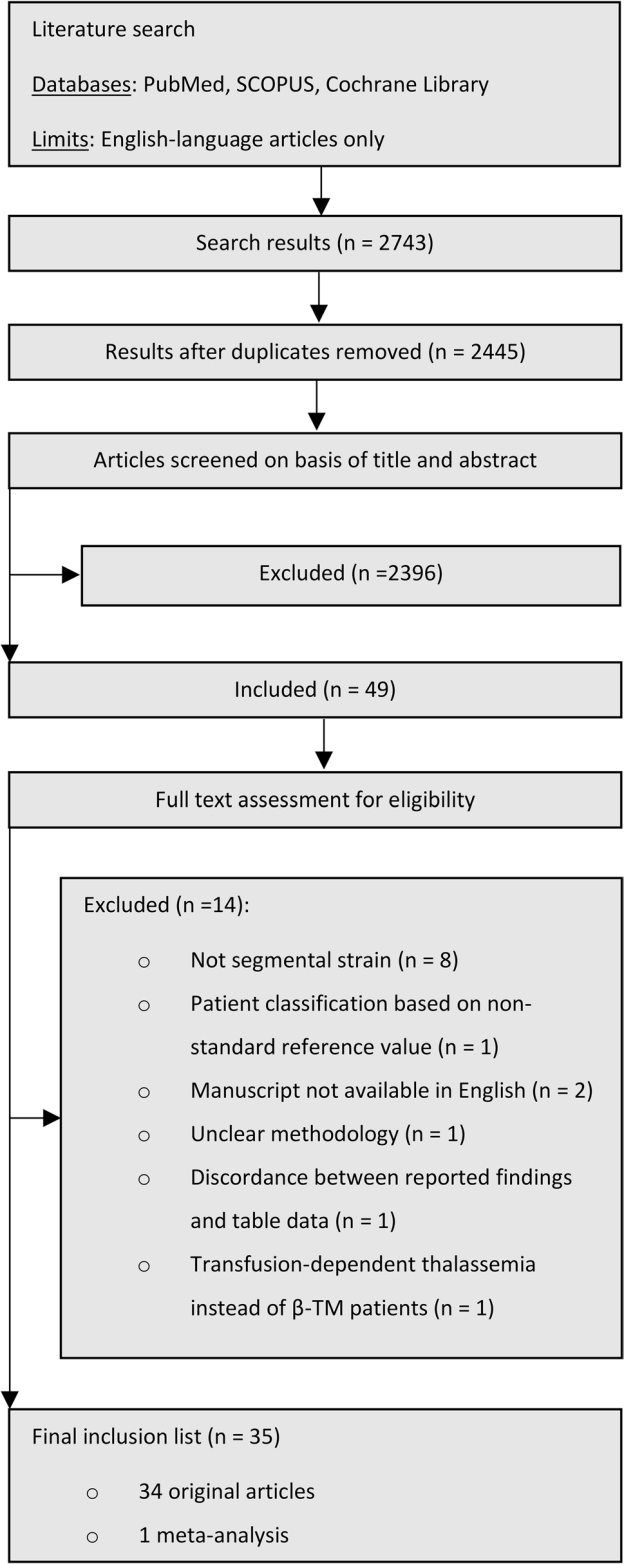
PRISMA flow chart

The PRISMA flow chart is depicted in Fig. 1. After the initial search through the electronic databases, 2743 articles emerged. A total of 298 duplicated articles were excluded and the remaining 2445 articles were screened on basis of title and abstract by two review authors (D.P, Y.D.). A total of 2396 articles were excluded as being irrelevant and the remaining 49 articles were thoroughly reviewed in full text. A total of 14 articles were eliminated for not fulfilling the inclusion criteria. Specifically, eight studies [14–21] were excluded for evaluating only segmental (but not global) ventricular strain, one study [22] for using non-standard reference value for patient classification (specifically, authors used T2* equal to 25 ms instead of the universally accepted cut-off value of 20 ms for MIO characterization), two studies [23, 24] with manuscripts in non-English language, one study [25] with unclear methodology, a study [26] reporting discordant results to the presented raw data on the corresponding tables and finally a study [27] on transfusion dependent thalassemia patients (being not equivalent to β-TM patients). A total of 35 articles (34 original articles and one meta-analysis) where qualified for the final inclusion list, which is displayed on Table 1. We decided to include the meta-analysis by Attar et al. [28], as it included supplementary data that were not available in the original articles. However, it should be noted that Attar et al. had included 11 studies regarding speckle tracking echocardiography alongside with 3 non-relevant studies (one implementing Feature Tracking—Cardiac Magnetic Resonance Imaging method and two studies without evaluating myocardial deformation).

**Table 1:** Characteristics of the eligible studies

Study	Country	Study type	Echo unit	Echo timing	Speckle tracking method	Magnetic Scanne	CMR timing	CMR method	Hemoglobin (g/dL)	Exclusion criteria
Cheung [2010] [44]	China	Cross-sectional, Case–control	GE Vivid 7	within 2 weeks post transfusion	2D: LV-GLS (A4C), LV-GRS and LV-GCS (PSAX)	Siemens Sonata 1.5 T	same as echo	T2*	NA	Valvular heart disease
Garceau [2011] [57]	Canada	Cross-sectional, Case–control	Siemens Medical System	NA	2D: LV-GLS (A4C, A3C, A2C), LV-GCS (PSAX)	Siemens Avanto 1.5 T	NA	T2*	NA	Arrhythmias, significant epicardial CAD, hypertension, significant valvular disease, LBBB
Piccione [2013] [48]	Italy	Cross-sectional, Case–control	MyLab 60	within 1 week post transfusion	2D: LV-GLS (A4C, A2C), LV-GRS and LV-GCS (PSAX)	NA	NA	T2*	9.7 ± 0.52 (pre-transfusion) and 12.9 ± 0.48 (post-transfusion)	T2* < 20 ms, arterial hypertension, diabetes mellitus, hypercholesterolemia, CAD, carotid atherosclerosis, > mild valvular heart disease, atrial fibrillation, smoking
Karamanou [2013] [52]	Greece	Cross-sectional	GE Vivid 7	4th day post-transfusion	2D: LV-GLS (A4C), LV-GRS and LV-GCS (PSAX), LASr and LAScd and LASct (A4C)	-	-	-	9.87 ± 0.5	Not in sinus rhythm, LVEF > 55%, history of CAD, mitral or aortic valve disease, congenital disease, pericardial disease, renal / thyroid / liver dysfunction, cor pulmonale
Chen [2015] [59]	Taiwan	Cohort, Case–control	Hewlett-Packard Sonos 5500	NA	2D: LV-GLS (A4C, A2C), LV-GRS and LV- GCS (PSAX)	-	-	-	NA	None
Li [2016] [45]	China	Cross-sectional, Case–control	Toshiba Artida	within 2 weeks post transfusion	3D: LV-GLS	Siemens Sonata 1.5 T	NA	T2*	NA	Arrhythmias
Hanneman [2016] [58]	Canada	Cross-sectional, Case–control	GE E9	NA	2D: LV-GLS (A4C, A3C, A2C)	Siemens Avanto 1.5 T	16 days (median)	T2*, unenhanced T1, EC V, LGE	11.81 ± 1.33	< 18 years old, claustrophobia, blood transfusion < 24 h, GFR < 30 ml/min, MRI contraind ication
Emre Ari [2017] [55]	Turkey	Crosssectional, Case–control	Philips iE33	Post transfusion	2D: LV-GLS (A4C, A2C), LV-GCS (PSAX)	Siemens Symphony	NA	T2*	12.4 ± 1.4 (post transfusion)	LV dysfunction, congenital or valvular heart disease, active infectious disease
Parsaee [2017] [37]	Iran	Crosssectional, Case–control	Philips iE33	NA	2D: LV-GLS (A4C, A3C, A2C), LV-GCS (PSAX)	NA	NA	T2*	9.3 ± 1.1	hypertension, diabetes mellitus, symptoms of heart failure, LV dysfunction, > mild valvulopathy, atrial fibrillation or flutter, ischemic heart disease, pulmonary hypertension
Di Odoardo [2017] [50]	Italy	Crosssectional, Case–control	GE Vivid 7	within 2 weeks post transfusion	2D: LV-GLS (A4C, A2C), LV-GRS and LVGCS (PSAX)	Siemens Avanto 1.5 T	recent	T2*	9.5 ± 0.6 (mean pre-transfusion level)	None
Pizzino [2017] [49]	Italy	Crosssectional	MyLAbalpha	1 week post transfusion	2D: LV-GLS (A4C, A3C, A2C) and LVGCS (PSAX)	GE Excite HDxt 1.5 T	same as echo	T2*, GH-T2*, LGE	9.3 ± 1.3	None
Poorzand [2017] [38]	Iran	Crosssectional	Philips iE33	within 2 weeks post transfusion	2D: LV-GLS (A4C, A3C, A2C), LASr and RASr (A4C)	Siemens Avanto 1.5 T	same as echo	T2*	NA	cardiovascular disease, risk factors for atherosclerosis
Parsaee [2018] [39]	Iran	Crosssectional	Philips iE33	10 days post transfusion	2D: LV-GLS (A4C, A3C, A2C)	Siemens Avanto 1.5 T	same as echo	T2*	9.18 ± 1.04	None
El Razaky [2019] [29]	Egypt	Crosssectional, Case–control	GE Vivid 7 / Vivid 9	before transfusion	3D: LV-GLS, LV-GRS and LV-GCS (PSAX)	-	-	-	7.85 ± 1.7	congenital or acquired heart disease
Cheung [2019] [46]	China	Crosssectional, Case–control	GE Vivid E95	within 2 weeks post transfusion	2D: LV and RV GLS (A4C), LV-GRS and LV-GCS (PSAX), LAS and RAS (positive, negative, total) (A4C)	NA	Most recent	T2*	NA	< 5 years old
Abtahi [2019] [40]	Iran	Crosssectional	GE Vivid 9	NA	2D: LV-GLS (A4C, A3C, A2C)	Philips 1.5 T	same week as echo	T2*	> 9.5	Cardiovascular disease, valvular heart disease, arrhythmias, endocrine diseases, hypertension, smoking, treatment with cardiovascular effects, LV dysfunction
El-Shanshory [2019] [30]	Egypt	Cohort, Case–control	GE Vivid 7	Not standard (Hb range 5.6 – 10.6)	2D: LV-GLS (A4C, A3C, A2C)	-	-	-	8.27 ± 1.31	Congenital or rheumatic heart disease, cardiomyopathies, CAD, systemic diseases
Parsaee [2019] [41]	Iran	Crosssectional	Philips Koninklijke	within 1 week post transfusion	2D: LV-GLS (A4C, A3C, A2C)	NA	within 3 days	T2*	9.58 ± 1.39	Not in sinus rhythm, reduced LVEF, valvular heart disease, pulmonary arterial hypertension
Hassan [2019] [60]	Iraq	Crosssectional, Case–control	GE	NA	2D: LV-GLS (A4C, A3C, A2C)	–	-	-	NA	None
Vlachou [2020] [53]	Greece	Crosssectional	GE Vivid 7 / Vivid S7	within 3 days before transfusion	2D: LV-GLS (A4C, A3C, A2C), LASr (A4C, A3C, A2C)	NA	Same as echo	T2*	10.2 ± 0.7	Systolic heart failure, CAD, atrial fibrillation, significant mitral or aortic valve disease, congenital heart disease, severe renal or liver dysfunction, thyroid disease, malignancies, electrolyte imbalance, acute or chronic inflammation diseases
El-Shanshory [2020] [31]	Egypt	Crosssectional, Case–control	GE Vivid 7	NA	2D: LV-GLS (A4C, A3C, A2C), GCS and LV-GRS (PSAX)	GE SIGNA EXCITE 1.5 T	within 10 days post transfusion	T2*	7.12 ± 0.9	congenital, rheumatic heart disease, cardiomyopathies, CAD, systemic diseases
Nadar [2020] [61]	Oman	Crosssectional, Case–control	GE Vivid 9	NA	2D: LV-GLS (A4C, A3C, A2C)	Siemens 1.5 T	within 2 months post echo	T2*	> 9.0	None
AbdelMassih [2020] [32]	Egypt	Crosssectional, Case–control	GE Vivid 7 / Vivid 9	NA	2D: LV-GLS (A4C, A3C, A2C), endocardial and epicardial strain (MLSD-STE)	Siemens 1.5 T	NA	T2*	9.3 ± 1.2	Congenital or rheumatic heart disease, present or past history of heart failure
Parsaee [2020] [42]	Iran	Crosssectional	Philips EPIQ 7	> 4 days post transfusion	2D: LASr (A4C)	GE Cvi 1.5 T	NA	T2*	10.4 [9.5—11.5]	< 18 years old
Barbero [2021] [51]	Italy	Crosssectional	GE Vivid 7	4–9 days post transfusion	2D: LV-GLS (A4C, A3C, A2C)	NA	Within 12 months	T2*	10.67 ± 0.65	Heart failure, pulmonary hypertension, valvular heart disease, arrhythmias, LVEF < 55%, pregnancy, < 18 years old, CAD
Nashat [2021] [33]	Egypt	Crosssectional, Case–control	GE Vivid E9	within 5 days post transfusion	2D: LV-GLS (A4C, A3C, A2C), RV-GLS (A4C)	-	-	-	7.87 ± 0.88	> 18 years old, structural heart disease, LVEF < 55%, pulmonary arterial hypertension, arrhythmia, diabetes mellitus, hypertension, renal and liver disorders, malignancies
El-Azm [2021] [34]	Egypt	Crosssectional, Case–control	Philips EPIQ 7	NA	2D: LV-GLS (A4C, A3C, A2C), LV-GCS (PSAX)	-	-		8.27 ± 1.23	> 18 years old
Sayed [2021] [35]	Egypt	Crosssectional, Case–control	Philips	NA	2D: LV-GLS (A4C, A3C, A2C)	-	-	-	9.7 ± 0.6	structural heart disease, smoking, hypertension, diabetes mellitus, dyslipidemia, obesity, history of heart failure due to non-iron overload causes
Okay [2021] [56]	Turkey	Crosssectional	GE Vivid E9	NA	2D: LV-GLS (A4C, A3C, A2C)	Siemens Aera 1.5 T	NA	T2*	NA	arrhythmias, hypertension, > mild valvular disease, congenital heart disease
Fattahi [2021] [43]	Iran	Cross-sectional	Philips EPIQ 7	NA	2D: LV-GLS (A4C, A3C, A2C), LVGCS	Siemens Avanto 1.5 T	same day	T2*	9.41 ± 0.96	hypertension, cardiac symptoms or disease, LVEF < 50%, > mild valvulopathy, atrial fibrillation or flutter, renal disease, smoking
See [2022] [47]	China	Cross-sectional	GE Vivid E95	within 2 weeks post transfusion	(PSAX), RVGLS (A4C) 3D: LV-GLS, LVGCS	Siemens Magneton 1.5 T	within 1.4 ± 0.5 years	T1 mapping, T2*	NA	None
Eroglu [2022] [8]	Turkey	Cross-sectional, Case–control	Philips iE33	before transfusion	2D: LV-GLS (A4C), LAS (positive, negative, total) (A4C)	Philips Ingenia 1.5 T	same day	T2*	9.03 [7.54—9.7]	congenital or acquired heart disease, chronic kidney disease, hepatic failure, autoimmune disease, infectious disease, arterial hypertension, obesity
Patsourakos [2022] [54]	Greece	Cross-sectional, Case–control	Philips EPIQ 7C	within 2 weeks post transfusion	3D: LV-GLS, LVGCS, LV-GRS	Siemens 1.5 T	within 12 months	T2*	9.33 ± 1.17	None
Elhawary [2022] [36]	Egypt	Cross-sectional, Case–control	GE Vivid 7	NA	2D: LV-GLS (A4C, A3C, A2C), LASr and LAScd and LASct (A4C)	-	-	-	7.23 ± 1.35	clinically evident cardiac manifestations
Attar [2022] [28]	-	Meta-analysis	-	-	2D: RV-GLS (A4C)	-	-	-	-	-

*A2C* apical 2 chambers view, *A3C* apical 3 chambers view, *A4C* apical 4 chambers view, *ECV* extracellular volume fraction, GH-T2* global heart T2*, *LAScd* left atrial strain at conduit phase, *LASct* left atrial strain at contraction phase, *LASr* left atrial strain at reservoir phase, *LGE* late gadolinium enhancement, *LV-GCS* left ventricular global circumferential strain, *LV-GLS* left ventricular global longitudinal strain, *LV-GRS* left ventricular global radial strain, *MLSD-STE* myocardial layer strain speckle tracking echocardiography, *NA* not available, *PLAX* parasternal long axis view, *PSAX* parasternal short axis view, *RASr* right atrial strain at reservoir phase, *RV-GLS* right ventricular global longitudinal strain

### Study characteristics

Out of the 34 selected studies, eight were conducted in Egypt [29–36], seven in Iran [37–43], four in China [44–47], four in Italy [48–51], three in Greece [52–54], three in Turkey [8, 55, 56], two in Canada [57, 58], one in Taiwan [59], one in Iraq [60] and one in Oman [61]. Thirty-two studies were cross-sectional and two were cohort [30, 59]. The majority of them (33 out of 34) were observational and one was experimental [30]. The latter consisted of GLS calculation before and after spirulina treatment. Only the baseline GLS measurements were taken into consideration for the purposes of the present review. Exclusion criteria greatly varied among the selected studies (Table 1). Most studies excluded patients with heart failure, left ventricular dysfunction, moderate or severe valvular heart disease, atrial fibrillation or atrial flutter. Twelve studies had no exclusion criteria. Three studies included only patients older than 18 years, while two other studies included only patients with age below 18 years. Ten out of thirty-four studies had a mean patient age below 18 years. Twenty-five studies also included CMR imaging for T2* calculation. Additionally, one of these studies also included unenhanced T1 and LGE [58], one included T1 mapping [47] and another one calculated not only mid-septal T2*, but Global-Heart T2* (GH-T2*) and LGE as well [49]. All study characteristics are summarized in Table 1.

### Patient characteristics

A total of 1592 β-TM patients were enrolled in the selected studies. Twenty-nine studies included exclusively β-TM patient groups, while the rest studies enrolled mixed patient groups (three of them [39, 42, 43] included β-TM and β-thalassemia intermedia (β-TI) patients, one [51] included β-TM, β-TI and sickle-cell disease (SCD) patients and one [57] β-TM and Blackfan-Diamond anemia patients). Moreover, three studies enrolled two separate patient groups (two of them [35, 61] included β-TM and β-TI patients and the third [32] β-TM and SCD patients). Twenty-two studies also enrolled a control group for comparison in terms of myocardial deformation. A total of 776 control subjects were enrolled in the selected studies. Of note, CMR imaging was performed only in patient groups. Without exception, all the selected studies reported that patients and controls did not differ in terms of sex or age.

### Left Ventricular Global Longitudinal Strain (LV-GLS)

Thirty-two of the 34 selected studies have evaluated LV-GLS (Table 2). Mean LV-GLS value in patient group was reported in 28 of them, while in the remaining studies, LV-GLS data were only available in MIO and non-MIO patient subgroups. As depicted on Table 1, there is heterogeneity in the echocardiographic speckle tracking methods that were applied. Specifically, 28 studies used 2D speckle tracking echocardiography (2D-STE), 4 studies used 3D speckle tracking echocardiography (3D-STE), while in one of them, both 3D and 2D-STE were applied. Regarding 2D-STE for LV-GLS estimation, image acquisition exclusively from A4C view was used in 4 studies, a combination of images from A4C and A2C views in 4 studies, while in the remaining ones, LV-GLS was calculated from all A4C, A3C and A2C views. Interestingly, a different approach of STE is the assessment of myocardial layer strain (MLSD-STE) as AbdelMassih et al. used [32]. Nine studies evaluated LV-GLS among β-TM patients of mean age below 18 years. Twenty-one studies also evaluated LV-GLS in a control group.

**Table 2:** Left ventricular strain studies

Study	Subjects	N	Male (%)	Age (years)	MIO(%)	Mean T2* (ms)	GLS (%)	GCS (%)	GLS in non-MIO (%)	GRS (%)	GLS in MIO (%)	GCS in MIO (%)	GCS in non-MIO (%)	GRS in MIO (%)	GRS in non-MIO (%)	T2* correlation	GLS cutoff value for MIO prediction
Cheung [2010] [44]	β-TM	42	42.9	24.4 ± 6.4	NA	NA	-17.8 ± 3.8	-14.2 ± 3.1	NA	48.3 ± 13.9*	NA	-	-	-	-	-	-
	Controls	38	50.0	22.4 ± 4.5	-	-	-19.2 ± 4.0	-14.4 ± 4.4	-	38.1 ± 12.2	-	-	-	-	-	-	-
Garceau [2011] [57]	β-TM + Blackfan—Diamond	40 + 5	54.0	34.4 ± 11	48.8	23 ± 13	-18.0 ± 3.0	-22.0 ± 5.0	-20.0 ± 4.0	-	-16.0 ± 3.0**	-20.0 ± 4.0**	-25.0 ± 4.0	-	-	GLS (r = -0.65), GCS (r = -0.39), *p* < 0.05	< -17% (76% sensitivity, 88% specificity)
	Controls	18	NA	NA	-	-	-20.0 ± 3.0	-23.0 ± 5.0	-	-	-	-	-	-	-	-	-
Piccione [2013] [48]	β-TM	32	28.1	35.3 ± 7.7	0.0	37.7 ± 5.5	-17.9 ± 3.5*	-20.5 ± 5.1	-	36.7 ± 8.2	-	-	-	-	-	GLS (r = -0.53, *p* = 0.001)	-
	Controls	33	39.4	34.9 ± 6.1	-	-	-24.3 ± 3.4	-22.4 ± 4.1	-	38.3 ± 5.8	-	-	-	-	-	-	-
Karamanou [2013] [52]	β-ΤΜ	88	43.2	36 ± 8.2	-	-	-18.7 ± 3.3	-17.3 ± 3.7	-	39.8 ± 13	-	-	-	-	-	-	-
Chen [2015] [59]	β-TM	37	56.8	24.2 ± 5.5	-	-	-16.6 ± 3.0*	-22.7 ± 4.8*	-	31.7 ± 11.6*	-	-	-	-	-	-	-
	Controls	40	55.0	24.0 ± 4.5	-	-	-20.2 ± 2.0	-27.1 ± 3.5	-	43.8 ± 12.3	-	-	-	-	-	-	-
Li [2016] [45]	β-TM	24	50.0	29.3 ± 5.2	25.0	32.7 ± 16.7	28.0 ± 7.4*	-	-	-	-	-	-	-	-	3D GLS (r = 0.74, p < 0.001)	-
	Controls	22	40.9	29.6 ± 5.8	-	-	41.5 ± 6.9	-	-	-	-	-	-	-	-	-	-
Hanneman [2016] [58]	β-TM	30	53.3	34.6 ± 9.5	63.3	28.2 ± 7.6	-19.2 ± 2.4	-	-	-19.4 ± 1.8	-18.8 ± 3.5	–	-	–	-	T1	-
	Controls	10	50.0	31.5 ± 4.4	-	-	-20.9 ± 1.8	-	-	-	-	-	-	-25.1 ± 5.8	-	(r = 0.874, *p* < 0.001)	-
Emre Ari [2017] [55]	β-TM	30	50.0	14.6 ± 3.6	33.3	23.9 ± 10.6	-21.9 ± 2.9	-	-23.0 ± 6.4*	-19.7 ± 3.1**	-23.1 ± 2.2	-	-19.0 ± 5.7**	-	-	-	-
	Controls	30	56.6	13.8 ± 2.2	-	-	-22.6 ± 2.6	-	-26.8 ± 4.6	-	-	-	-	-	-	-	-
Parsaee [2017] [37]	β-TM	55	49.1	27.5 ± 8.8	27.3	23.5 ± 9.8	-20.9 ± 1.9*	-	-24.8 ± 2.5	-	-	-	-	-	-	-	-
	Controls	18	44.4	29.0 ± 6.0	-	-	-22.2 ± 1.0	-	-23.7 ± 2.5	-	-	-	-	-20.1 ± 4.4	-	-	-
Di Odoardo [2017] [50]	β-TM	55	38.2	36.6 ± 7.0	38.0	26.6 ± 14.1	-19.0 ± 2.9	34.6 ± 12.3*	-19.5 ± 4.0*	-18.8 ± 2.6	-19.2 ± 3.0	32.2 ± 12.9	-18.7 ± 3.2	-	36.1 ± 11.9	-	-
	Controls	20	55.0	42.0 ± 7.6	-	-	-19.0 ± 2.7	43.2 ± 2.7	-25.9 ± 2.0	-	-	-	-		-	-	-
Pizzino [2017] [49]	β-TM	28	53.6	37.4 ± 10	21.4	40.5 [32-44]	-20.6 ± 2.8	-19.5 ± 2.6	42.4 ± 8.3	-18.3 ± 2.0**	-21.3 ± 2.7	-17.9 ± 2.2	-19.9 ± 2.5	39.4 ± 10.5	43.3 ± 7.7	GLS (r = -0.41, *p* = 0.031)	< -19.5% (83% sensitivity, 77.3% specificity)
Poorzand [2017] [38]	β-TM	44	52.3	23.5 ± 6.2	31.8	NA	NA	-	-	-18.2 ± 3.4**	-20.3 ± 1.7	-	-	-	-	GLS (r = -0.42, *p* = 0.001)	< -17.5% (43.8% sensitivity, 100% specificity)
Parsaee [2018] [39]	β-TM + β-thalassemia intermedia	75 + 47	45.1	30.8 ± 9.4	33.6	NA	NA	-	-	-17.0 [-16 to—19]**	-19.0 [-18 to -20]	-	-	-	-	NA	< -18.5% (73% sensitivity, 63% specificity)
El Razaky [2019] [29]	β-TM	100	49.0	8.0 ± 3.8	-	-	-14.9 ± 12.1*	-8.1 ± 3.8*	33.1 ± 10.6*	-	-	-	-	-	-	-	-
	Controls	100	NA	7.9 ± 3.6	-	-	-19.1 ± 1.5	-16.3 ± 1.3	37.3 ± 4.2	-	-	-	-	-	-	-	-
Cheung [2019] [46]	β-TM	38	50.0	34.5 ± 10.7	10.5	35.9 ± 10.1	-17.5	NA	NA	-	-	-	-	-	-	-	-
	Controls	43	41.9	30.3 ± 12.6	-	-	-17.5	NA	NA	-	-	-	-	-	-	-	-
Abtahi [2019] [40]	β-TM	52	55.8	23.7 ± 5.0	42.3	19.8 ± 10.3	-19.4 ± 3.2	-	-	-17.6 ± 2.6**	-21.6 ± 2.7	-	-	-	-	GLS (r = -0.6, *p* < 0.001)	< -19.5% (82.1% sensitivity, 86.4% specificity)
El- Shanshory [2019] [30]	β-TM	60	50.0	10.2 ± 3.0	-	-	-21.7 ± 4.6*	-	-	-	-	-	-	-	-	-	-
	Controls	30	43.3	9.5 ± 2.6	-	-	-23.9 ± 2.1	-	-	-	-	-	-	-	-	-	-
Parsaee [2019] [41]	β-TM	45	44.0	32.0 ± 7.5	42.2	NA	NA	-	-	-21.7 ± 1.7	-22.4 ± 2.3	-	-	-	-	-	-
Hassan [2019] [60]	β-TM	20	NA	10.0 ± 3.8	-	-	-23.6 ± 3.0	-	-	-	-	-	-	-	-	-	-
	Controls	20	NA	11.0 ± 1.7	-	-	-22.0 ± 1.8	-	-	-	-	-	-	-	-	-	-
Vlachou [2020] [53]	β-TM	50	50.0	37.6 ± 9.1	NA	33.1 ± 9.6	-20.0 ± 2.7	-	-	-	-	-	-	-	-	-	-
El-Shanshory [2020] [31]	β-ΤΜ	100	40.0	11.0 ± 3.8	32.0	35.6 ± 26.8	-21.3 ± 2.5	-9.1 ± 5.3*	31.7 ± 11.0*	-21.4 ± 2.1	-21.2 ± 2.7	-10.1 ± 4.1	-8.6 ± 5.8	34.1 ± 10.5	30.5 ± 11.1	-	-
	Controls	40	40.0	10.6 ± 3.8	-	-	-22.3 ± 1.8	-16.1 ± 1.3	36.9 ± 4.5	-	-	-	-	-	-	-	-
Nadar [2020] [61]	β-TM	84	42.8	26.3 ± 6.1	27.4	31.8 [16.0 to 39.3]	-19.1 ± 2.7*	-	-	-18.6 ± 2.4	-19.2 ± 2.8	-	-	-	-	-	-
	β-thalassemia intermedia	17	41.1	30.0 ± 10.0	0.0	37.0 ± 5.7	-19.8 ± 2.9	-	-	-	-	-	-	-	-	-	-
	Controls	53	NA	NA	-	-	-20.4 ± 2.8	-	-	-	-	-	-	-	-	-	-
AbdelMassih [2020] [32]	β-TM	40	50.0	11.0 ± 3.0	NA	16.6 ± 1.8	-15.0 ± 1.6*	-	-	-	-	-	-	-	-	-	-
	sickle-cell	40	50.0	10.0 ± 4.0	NA	25.5 ± 2.2	-15.0 ± 1.2*	-	-	-	-	-	-	-	-	-	-
	Controls	40	NA	10.0 ± 3.0	-	-	-21.5 ± 1.9	-	-	-	-	-	-	-	-	-	-
Barbero [2021] [51]	β-TM + β-thalassemia intermedia + sickle cell	58 + 3 + 1	50.0	40.4 ± 7.9	13.8	39.7 [33.3—45.7]	-18.4 ± 3.6	-	-	-19.6 ± 2.6	-20.4 ± 3.7	-	-	-	-	-	< -17.5% (80% sensitivity, 96% specificity)
Nashat [2021] [33]	β-TM	50	56.0	14.8 ± 4.7	-	-	-21.3 ± 3.5*	-	-	-	-	-	-	-	-	-	-
	Controls	30	36.7	13.8 ± 3.3	-	-	-24.9 ± 1.0	-	-	-	-	-	-	-	-	-	-
El-Azm [2021] [34]	β-TM	31	77.4	10.3 ± 3.1	-	-	-17.7 ± 4.1*	-20.0 ± 6.2*	-	-	-	-	-	-	-	-	-
	Controls	31	64.5	9.3 ± 2.4	-	-	-20.8 ± 2.7	-23.4 ± 6.2	-	-	-	-	-	-	-	-	-
Sayed [2021] [35]	β-TM	20	45.0	27.0 ± 6.4	-	-	-21.7 ± 1.9*	-	-	-	-	-	-	-	-	-	-
	β-thalassemia intermedia	20	55.0	23.4 ± 6.2	-	-	-19.4 ± 1.7*	-	-	-	-	-	-	-	-	-	-
	Controls	40	45.0	24.5 ± 7.5	-	-	-24.7 ± 1.8	-	-	-	-	-	-	-	-	-	-
Okay [2021] [56]	β-TM	45	46.7	31.6	26.7	NA	NA	-	-	-20.7 [-12.8 to -26.5]	-19.9 [-12.0 to -27.1]	-	-	-	-	-	< -20.5% (83% sensitivity, 54% specificity)
Fattahi [2021] [43]	β-TM + β- thalassemia intermedia	39 + 9	54.2	31.0 ± 8.0	33.3	24.2 [3-43]	-22.0 ± 4.6	-30.1 ± 6.5	-	-21.6	-22.2	-28.4	-31.0	-	-	3D LVGLS (r = -0.5), 3D LVGCS (r = -0.49), *p* < 0.05	< -22.3%
See [2022] [47]	β-TM	34	47.1	35.5 ± 9.2	8.8	36.4 ± 8.7	-17.4 ± 2.3	-	-	-	-	-	-	-	-	-	-
Eroglu [2022] [8]	β-TM	40	70.0	17.3 ± 3.1	22.5	28.5 ± 11.5	-25.7 [-18.7 to—29.4]*	-26.4 ± 2.2*	42.6 [33.5 to 49.4]*	-25.7 [- 8.7 to—29.4]	-24.6 [-21.1 to—27.7]	-26.3 [-20.6 to—31.0	-26.0 [-22.5 to—29.4]	42.7 [35.2 to 49.4]	41.2 [33.5 to 46.6]	-	-
	Controls	40	67.7	17.2 ± 3.1	-	-	-26.5 [-22.3 to—28.9]	-28.2 ± 1.7	45.4 [36.6 to 48.8]	-	-	-	-	-	-	-	-
Patsourakos [2022] [54]	β-TM	56	50.0	39.3 ± 9.0	16.1	31.8 ± 10.7	-20.4 ± 2.0*	-	-	-19.0 ± 2.6	-20.7 ± 2.6	-	-	-	-	-	-
	Controls	30	36.7	36.7 ± 10.2	-		-21.9 ± 2.0	-	-	-	-	-	-	-	-	-	-

*GCS* left ventricular global circumferential strain, *GLS* left ventricular global longitudinal strain, *GRS* left ventricular global radial strain, *MIO* myocardial iron overload

Data are presented as mean (SD), median [IQR], or N (%).

*. Statistically significant difference between patient and control groups.

**. Statistically significant difference between MIO and non-MIO groups.

Mean LV-GLS ranged from -14.9 ± 12.1% to -28.0 ± 7.4% among patients. After comparing patient and controls, LV-GLS was found significantly worse in 13 of 21 studies (Table 2, significant difference is marked with * symbol). Of note, significant difference in LV-GLS was reported in 6 of 9 studies with mean patient age below 18 years. Moreover, 3D-STE that was implemented in 3 case–control studies, revealed worse LV-GLS in patient group compared to controls. Parsaee et al. [41] performed exercise stress echocardiography and compared LV-GLS measurements at baseline and at peak exercise. The authors reported that MIO patients had declining GLS at peak exercise compared to baseline, while non-MIO patients found to have improved peak exercise GLS compared to baseline. Finally, according to Chen et al. [59], LV-GLS values less negative than -15.48% could predict adverse clinical events (arrhythmia, heart failure, death) in β-TM patient groups, with LV-GLS being the only independent prognostic index for adverse clinical events, after adjusting for age, sex, serum ferritin level and LV mass index (HR: 1.54, 95% CI: 1.02–2.53).

### Left Ventricular Global Circumferential Strain (LV-GCS)

LV-GCS was evaluated in 15 studies and corresponding data were available in 14 of them (Table 2). LV-GCS ranged from -8.1 ± 3.8% to -30.1 ± 6.5%. In 11 of these studies, LV-GCS was also evaluated in controls, while 5 studies reported LV-GCS values among patient groups of mean age below 18 years. LV-GCS was reported being significantly worse in patients compared to controls in 7 of 11 case–control studies. Of note, LV-GCS significantly differed in all case–control studies with mean patient age below 18 years. According to a univariable model by Chen et al. [59], LV- GCS values less negative than -21.31% could predict adverse clinical events (HR: 1.27, 95% CI: 1.09–1.48) among β-TM patients.

### Left Ventricular Global Radial Strain (LV-GRS)

LV-GRS was evaluated in 9 studies and in 7 of them a comparison was made between patients and control (Table 2). Mean LV-GRS among patient groups ranged from + 31.7 ± 11.0% to + 48.3 ± 13.9%, while in 3 of these studies the mean patient age was below 18 years. LV-GRS was found to be worse among patients in 6 out of 7 case–control studies. Of note, in all studies with mean patient age below 18 years, LV-GRS differed significantly between patients and controls. According to Chen et al. [59], LV-GRS less than 26.67% could predict adverse clinical events (HR: 1.14, 95% CI: 1.05–1.25) among β-TM patients.

### Right ventricular global longitudinal strain (RV-GLS)

RV-GLS was evaluated in 4 of the selected studies and in 3 of them a control group has been included (Table 3). RV-GLS was found being worse in patients compared to controls in 2 of the 3 case–control studies. Of note, the studies with significant difference in RV-GLS consisted of patients with mean age below 18 years, while the third study enrolled older patients. CMR imaging was implemented only in 2 of the 4 studies. A negative correlation was reported between T2* and RV-GLS (*r* = -0.25, *p* < 0.05) by Fattahi et al. [43], however MIO and non-MIO patient groups did not differ in terms of RV-GLS.

**Table 3:** Right ventricular strain studies

Study	Subjects	N	Male (%)	Age (years)	MIO (%)	Mean T2* (ms)	RV-GLS (%)	RVGLS in MIO (%)	RVGLS in non-MIO (%)	T2* correlation
Cheung [2019] [46]	β-TM	38	50.0	34.5 ± 10.7	10.5	35.9 ± 10.1	-18.5	-	-	-
	Controls	43	41.9	30.3 ± 12.6	-	-	-19.0	-	-	-
Nashat [2021] [33]	β-TM	50	56.0	14.8 ± 4.7	-	-	-21.7 ± 5.6*	-	-	-
	Controls	30	36.7	13.8 ± 3.3	-	-	-25.3 ± 2.3	-	-	-
Fattahi [2021] [43]	β-TM + β-thalassemia intermedia	39 + 9	54.2	31.0 ± 8.0	33.3	24.2 [3-43]	NA	-12.4	-13.8	RV-GLS (r = -0.25), *p* < 0.05
Elhawary [2022] [36]	β-TM	50	46.0	8.6 ± 4.8	-	-	-18.0 [-15.0 to—21.0]*	-	-	-
	Controls	50	52.0	8.0 ± 4.0	-	-	-23.0 [-20.0 to—25.0]	-	-	-

*RV-GLS* right ventricular global longitudinal strain, *MIO* myocardial iron overload

Data are presented as mean (SD), median [IQR], or N (%).

*. Statistically significant difference between patient and control groups.

### Atrial strain

Left atrial strain was evaluated in 7 of the selected studies (Table 4), while 2 of them also included right atrial strain calculation (Table 5). There has been heterogeneity in the reported atrial deformation indices. Specifically, 4 of the 7 studies reported atrial strain in terms of reservoir, conduit and contraction strain, while the remaining studies reported positive, negative and total strain. Only 2 studies have included a control group. The first one by Cheung et al. [46], reported that patients had worse positive and total atrial strain compared to controls, while Patsourakos et al. [54] found worse LASr and LAScd in patients compared to controls. A direct comparison between MIO and non-MIO patient subgroups was performed in 4 studies. In particular, Parsaee et al. [42] reported that LASr was lower in MIO patients compared to non-MIO, while Patsourakos et al. [54] reported that all atrial deformation indices were worse in MIO patients compared to non-MIO patients. On the contrary, no significant differences in terms of either LASr or RASr were found by Poorzand et al. [38] between MIO and non-MIO subgroups, while Cheung et al. [46] found no differences in either left or right atrial strain between these patient subgroups. A positive correlation between T2* and LASr was reported by both Parsaee et al. [42] and Patsourakos et al. [54] (r = 0.40, *p* < 0.001 and r = 0.35, *p* = 0.007 respectively).

**Table 4:** Left atrial strain studies

Study	Subjects	N	Male (%)	Age (years)	MIO (%)	Mean T2* (ms)	LASr (%)	LAScd (%)	LASct (%)	LAS positive (%)	LAS negative (%)	LAS total (%)	LASr in MIO (%)	LASr in non-MIO (%)	T2* correlation	LASr prediction
Karamanou [2013] [52]	β-ΤΜ	88	43.2	36 ± 8.2	-	-	32.7 ± 9.1	10.0 ± 4.6	-3.4 ± 1.6	-	-	-	-	-	-	LASr > 41.1% rules out E/e' > 8 (90.3% sensitivity, 81% specificity)
Poorzand [2017] [38]	β-TM	44	52.3	23.5 ± 6.2	NA	NA	-	-	-	-	-	-	39.5 ± 9.9	44.6 ± 10.1	-	-
Cheung [2019] [46]	β-TM	38	50.0	34.5 ± 10.7	10.5	35.9 ± 10.1	-	-	-	20.3 ± 5.3*	-9.2	29.5 ± 5.4*	-	-	-	-
	Controls	43	41.9	30.3 ± 12.6	-	-		–	-	23.1 ± 3.9	-9.4	32.5 ± 4.6	-	-	-	-
Vlachou [2020] [53]	β-TM	50	50.0	37.6 ± 9.1	NA	33.1 ± 9.6	33.8 ± 7.1	-	-	-	-	-	-	-	-	LASr < 31.5% predicts PACs > 24/d (83% sensitivity, 68% specificity)
Parsaee [2020] [42]	β-TM + β-TI	90	53.3	29.0 ± 6.0	31.0	24.9 [18.0 to 30.2]	-	-	-	-	-	-	32.0 [25.0 to 38.0]**	40.0 [33.0 to 44.0]	LASr (r = 0.40, *p* < 0.001)	-
See [2022] [47]	β-TM	34	47.1	35.5 ± 9.2	17.4	36.4 ± 8.7	-	-	-	19.6 ± 4.8	-9.1 ± 3.3	28.7 ± 4.8	-	-	-	-
Patsourakos [2022] [54]	β-TM	56	50.0	39.3 ± 9.0	16.1	31.8 ± 10.7	39.8 ± 13.0*	-27.1 ± 9.9*	-12.7 ± 5.8	-	-	-	24.7 ± 6.5**	42.2 ± 13.1	LASr (r = 0.347, *p* = 0.009), LASct (r = -0.359, *p* = 0.007)	-
	Controls	30	36.7	36.7 ± 10.2	-	-	49.8 ± 10.0	-34.6 ± 10.1	-15.1 ± 4.6	-	-	-	-	-	-	-

*E/e’* ratio between early mitral inflow velocity and mitral annular early diastolic velocity, *LAS* left atrial strain, *LAScd* left atrial strain at conduit phase, *LASct* left atrial strain at contraction phase, *LASr* left atrial strain at reservoir phase, *MIO* myocardial iron overload, *PACs* premature atrial contractions, *β-TI* β-Thalassemia intermedia

Data are presented as mean (SD), median [IQR], or N (%).

*. Statistically significant difference between patient and control groups.

**. Statistically significant difference between MIO and non-MIO groups.

**Table 5:** Right atrial strain studies

Study	Subjects	N	Male (%)	Age (years)	MIO (%)	Mean T2* (ms)	RASr (%)	RAS positive (%)	RAS negative (%)	RAS total (%)	RASr in MIO (%)	RASr in non-MIO (%)	T2* correlation
Poorzand [2017] [38]	β-TM	44	52.3	23.5 ± 6.2	NA	NA	NA	-	-	-	42.0 ± 9.1	45.4 ± 8.9	-
Cheung [2017]	β-TM	38	50.0	34.5 ± 10.7	10.5	35.9 ± 10.1	-	16.4 ± 5.0*	-10.3	26.7 ± 5.3*	-	-	-
	Controls	43	41.9	30.3 ± 12.6	-	-	-	19.9 ± 5.7	-10.3	30.2 ± 6.6	-	-	-

*RAS* right atrial strain, *RASr* right atrial strain at reservoir phase, *MIO* myocardial iron overload

Data are presented as mean (SD), or N (%).

*. Statistically significant difference between patient and control groups.

According to Karamanou et al. [52], LASr was found being worse among patients with E/e’ > 8 compared to patients with E/e’ < 8, while LASr values greater than 41.1% could rule out the presence of diastolic dysfunction (in particular E/e’ > 8) with a sensitivity of 90.3% and a specificity of 81%. Moreover, Karamanou et al. [52] reported a negative correlation between E/e’ and LASr (r = -0.36, *p* = 0.001). Vlachou et al. [53] reported that patients with increased number of premature atrial complexes (PACs) had worse LASr compared to patients with low number of PACs. The authors also reported that LASr was strongly associated with PACs > 24/d (OR = 0.89, CI 0.81–0.98, *p* = 0.03), while LASr values less than 31.5% could predict PACs > 24/d (83% sensitivity and 68% specificity). Of note, Vlachou et al. [53] followed Modin’s recommendation for left atrial strain evaluation from A4C, A3C and A2C views, as opposed to Badano’s recommendation for LASr evaluation from either A4C or a combination of A4C and A2C views [62, 63]. Additionally, Patsourakos et al. [54] reported all left atrial deformation indices being worse among patients with prior episodes of atrial fibrillation compared to patients with no history of atrial fibrillation.

Right atrial deformation was evaluated in a case–control study by Cheung et al. [46] that reported significant difference in the patient group compared to controls in terms of RASr and RAScd, but not RASct. The authors also reported a correlation between right and left atrial positive strain, between left and right atrial negative strain and finally between left and right atrial total strain. Neither Cheung et al. [46], nor Poorzand et al. [38] found any differences between MIO and non-MIO patient subgroups in terms of right atrial deformation.

It should be noted that all studies regarding atrial mechanics were performed in patient groups of mean age greater than 18 years.

### Myocardial Iron Overload (MIO)

CMR imaging was implemented in 25 of the 34 selected studies for myocardial iron status estimation. Patient groups were further divided into MIO and non-MIO subgroups based on a cut-off T2* value of 20 ms. Regarding left ventricular strain, MIO and non-MIO discrimination was made in 15 studies. LV-GLS was significantly worse in MIO subgroup compared to non-MIO subgroup in 6 of 15 studies, LV-GCS in 2 of 7 studies, while in 4 studies having calculated LV-GRS, no difference was reported in any of these. A correlation between T2* and LV-GLS was found in 7 studies. In 8 studies the corresponding authors reported a cut-off LV-GLS value that could predict a T2* value less than 20 ms, ranging from –

17% to -22.3%, with varying sensitivity and specificity (Table 2). In an interesting article, Hanneman et al. [58] implemented multiple CMR imaging techniques (native T1, T2*, Extracellular Volume Fraction (ECV)) among β-TM patients and reported that ECV correlated with “history low” T2*, but not with “same-day” T2*. Pizzino et al. [49] calculated not only the conventional T2* from the middle septum, but the global heart T2* (GH-T2*), with LV-GLS correlating with both these indices.

### Β-TM vs. other hemoglobinopathies

Both Nadar et al. [61] and Sayed et al. [35] evaluated LV-GLS in β-TM and β-TI patients. The former reported worse LV-GLS in β-TM patient group (-19.1 ± 2.7% vs 19.8 ± 2.9%, *p* = 0.03), while the latter in β-TI group (-21.7 ± 1.9% vs 19.4 ± 1.7%, *p* = 0.013).

AbdelMassih et al. [32] applied MLDS-STE in both β-TM and sickle-cell patients. The authors reported worse epicardial strain among β-TM patients compared to sickle-cell patients (-10.9 ± 2% vs -19.9 ± 1.7%, *p* < 0.01), as well as worse endocardial strain in sickle-cell patients compared to β-TM patients (-10.7 ± 1.6% vs -20.00 ± 1.7%, *p* < 0.01).

## Discussion

### Summary of evidence

The present systematic review summarizes the results of a total of 34 studies evaluating myocardial deformation using speckle tracking echocardiography in β-TM population groups that have been published until August 31^th^ 2022. Most studies reported statistically significant differences in terms of myocardial deformation between β-TM patients and healthy controls.

### Left ventricular strain

LV-GLS has been reported being worse in β-TM patients compared to controls in 13 of 21 case–control studies. It would be interesting to take a closer look on the 8 studies which failed to show a significant difference and consider them in terms of chronology, echocardiographic methodology, anemia state, myocardial iron status and sample size. These studies have been published before 2020, an observation that could lead to the assumption that latter studies having implemented improved versions of strain processing software could provide more accurate calculation of myocardial strain. LV-GLS was calculated from A4C, A3C and A2C views in 4 studies, while Cheung et al. [44], Emre Ari et al. [55], Di Odoardo et al. [50] and Cheung et al. [46] used either one or two apical echocardiographic views to calculate LV-GLS. According to the EACVI/ASE directives, all apical views are needed in order to accurately evaluate LV-GLS [64]. By comparison, among the 13 studies that showed difference in terms of LV-GLS, only two of them did not comply with the aforementioned directives.

Regarding the anemia state, 4 out the 8 studies did not provide data on hemoglobin concentration at the time of the examination. Hanneman et al. [58] and Emre Ari et al. [55] al reported relative preserved mean hemoglobin concentrations (11.81 and 12.4 g/dL respectively), while Di Odoardo et al. [50] reported mean pre-transfusion hemoglobin value equal to 9.5 g/dL. Only El-Shanshory et al. [31] have reported a relatively low mean hemoglobin concentration (7.12 g/dL). The effect of chronic anemia state on myocardial mechanics is yet undetermined. The ventricular remodeling and the myocardial oxygen supply–demand mismatch could result in impaired GLS, while the increased myocardial contractility due to compensatory cardiac output increase could improve GLS. The are scarce data in literature on this particular subject. In a study on 3D LV-GLS in patients with iron deficiency anemia by Zhou et al. [65], it has been reported that left ventricular strain was impaired in hemoglobin levels < 9 g/dL compared to patients with Hb > 9 g/dL. On the contrary, Cebeci et al. in a similar study [66] did not find significant difference in either 2D or 3D GLS among patient groups with Hb 7–9 g/dL, 9–12 g/dL and > 12 g/dL respectively. The authors reported that this discrepancy could be attributed to the difference in the average duration of anemia (6.5 years in the former study compared to 12 months in the latter). These findings should be interpreted with caution after taking into consideration not only the stable low hemoglobin concentrations in iron-deficiency anemia as opposed to the labile hemoglobin concentrations due to regular blood transfusions in β-TM, but the deleterious effect of excess iron on myocardial contractility among β-TM with MIO.

The percentage of MIO in patient groups ranged from 10.5% to 63.3%. Two of 8 studies did not provide data on MIO prevalence in patient group. The study by Piccione et al. [48] is unique in terms of exclusion of MIO patients, allowing the unveiling of the effect of anemia on myocardial strain without the concurrent effect of iron. The authors reported a significantly impaired LV-GLS in patients compared to controls, as well as a negative correlation between LV-GLS and T2*.

Finally, it would be worthwhile mentioning that half of these studies were underpowered. In particular, from a total of 16 groups included in these studies, 5 groups consisted of less than 30 participants. Garceau et al. [57], Hanneman et al. [58] and Di Odoardo et al. [50] enrolled control groups consisted of less than 20 subjects and in any case less than half the size of the corresponding patient groups. In 4 of these studies, patients had impaired LV-GLS compared to controls but this difference did not reach statistical significance. A possible explanation could be the relatively small groups, ranging from 10 to 40 participants in either patient or control group. In two studies there were similar LV-GLS values between patients and controls, while surprisingly enough, in the study by Hassan et al. [60], patients reportedly had better LV-GLS compared to controls. Unfortunately, due to insufficient supplementary data in that particular study regarding mean Hb level and the MIO prevalence in patient group, further interpretation is not feasible.

By comparison, among the 13 studies that have shown significant difference in terms of LV-GLS between patients and controls, the mean hemoglobin concentration was above 9.0 g/dL in 7 studies, between 8.0 and 9.0 g/dL in 2 studies, less than 8 g/dL in 2 studies, while no data were available in the remaining 2 studies. An interesting notice is that every study published since 2020 (apart from the study by El-Shanshory et al. [31] have shown significantly impaired LV-GLS in patients compared to controls. Regarding 2D LV-GLS methodology, image acquisition from all three apical views was used in 8 studies, while 2 studies used image acquisition from A4C and A2C views. No study calculated LV-GLS from a single A4C view, while all studies that implemented 3D speckle tracking technique reported significantly impaired 3D LV-GLS in patients compared to controls. In the study by Fattahi et al. [43] that 2D-STE was directly compared to 3D-STE, 3D-GLS values were more negative than 2D-GLS values, while a positive correlation was found between 2D-GLS and 3D-GLS. In 7 out of 13 studies there were no data regarding the prevalence of MIO, one study had totally excluded patients with MIO, while in the remaining 5 studies the prevalence of MIO ranged from 16.1% to 27.4%. Finally, considering sample size, the studies that revealed statistically significant difference in LV-GLS had larger groups. From a total of 26 groups (patients and controls) included in these studies, only 4 groups consisted of less than 30 participants.

Having taken all the aforementioned parameters into consideration, there are evidence indicating a trend of LV-GLS to be impaired among β-TM patients compared to controls. Based on the knowledge gained from the present study, some suggestions could be proposed. Future studies on this particular subject should not be underpowered, should implement either optimal 2D LV-GLS calculation or 3D speckle tracking technique. Finally, the echocardiographic study should not be performed either immediately before or after blood transfusion.

Regarding myocardial iron overload, it should be noted that in 13 of the 16 related studies, LV-GLS had lower values in MIO patients compared to non-MIO patients, although statistical significance was found only in 6 of them. To further clarify the effect of MIO on myocardial strain, these studies should be considered in terms of temporal association between echocardiographic examination and CMR imaging, the sample size and the variety of echocardiography vendors.

Echocardiographic examination and CMR imaging were performed on the same day in 7 LV-GLS related studies. A total of 10 studies had these examinations performed within a time frame of one week, while in 3 studies there was a time interval of a month or more between these exams. Finally, this particular time interval was not reported in 7 studies. Of note, out of the 6 studies that showed significant difference between MIO and non-MIO patients in terms of LV-GLS, 4 of them had the imaging examinations performed in less than a week apart, while in the remaining 2 studies the time interval was not reported.

The split of the patient groups into MIO and non-MIO subgroups inevitably has resulted into the generation of subgroups of small sample size, especially in studies with low prevalence of MIO. The only studies that reached a minimum of 30 patients in both MIO and non-MIO subgroups were those by Parsaee et al. [39] and El-Shanshory et al. [31]. Consequently, the results of t-test between these subgroups should be interpreted after careful consideration.

The wide range of LV-GLS values that could identify MIO could be attributed to the variety of echocardiographic units that were used, the temporal association of the imaging modalities, the variety of MIO prevalence or the hemoglobin concentration. Among the 8 studies that reported a LV-GLS value for MIO prediction, 6 different echocardiographic units have been used. These studies did not have significant differences regarding the speckle tracking method, the prevalence of MIO, the hemoglobin concentration or the temporal association between echocardiography and CMR imaging. Specifically, with no exception, LV-GLS was calculated by a combination of all three apical echocardiographic views. Mean hemoglobin value ranged from 9.18 to 10.67 g/dL, although in 3 out of the 8 studies no hemoglobin data were available. The echocardiographic examination and the CMR imaging were performed almost at the same time, with the exception of Barbero et al. [51] who performed them within 12 months and other 2 studies that the specific time interval is not reported. Finally, the MIO prevalence among these studies ranged from 13.8% to 48.8%.

In a similar fashion, LV-GCS was found being worse in patients in 7 of 11 case–control studies. Unlike LV-GLS, there was no heterogeneity in LV-GCS evaluation method, since it was calculated from PSAX view. The studies that did not report a significant difference in terms of LV-GCS in patient groups had either enrolled small control groups (Garceau et al. [57], Parsaee et al. [37] or had excluded patients with T2* < 20 ms (Piccione et al. [48]. All studies with patient age below 18 years, reported that LV-GCS was worse in patients compared to controls. Regarding myocardial iron overload, LV-GCS was found being worse in MIO compared to non-MIO patients only in 2 out of 7 related studies. It is considered that LV-GCS represents the systolic deformation of the subepicardial layer of the left ventricle, which is the earliest site of myocardial iron deposition [17, 67, 68], while LV-GLS represents the systolic deformation of the subendocardial layer that is primarily affected by chronic hypoxia as well as by iron deposition [17].

Similarly, LV-GRS was found to be lower in patients compared to controls in most of the related studies, as well as in every study with subjects of mean age below 18 years. In terms of myocardial iron overload, LV-GRS seems to be the less affected myocardial deformation index, as no study revealed differences between MIO and non-MIO patient subgroups. This observation is in concordance with the hypothesis that LV-GRS is related at a greater degree to left ventricular total mass rather than to myocardial iron load [44].

In conclusion, there is no strong evidence that left ventricular strain indices are reliable for MIO detection and consequently they cannot be proposed as alternatives to the gold standard method (CMR T2*). For further clarification, larger studies have to be designed with ideally equal size of MIO and non-MIO groups for increased statistical power. Nevertheless, there is sufficient evidence that myocardial deformation indices can be used to detect early left ventricular dysfunction in β-TM patients, that is considered multifactorial due to iron overload, chronic tissue hypoxia, individual susceptibility to iron toxicity, myocarditis, viral infections and immunodeficiency [69–72]. It has been reported that 10% of β-TM patients eventually develop heart failure without myocardial iron overload [73]. Nowadays, left ventricular deformation indices are being added to the routine cardiological assessment of a growing number of diseases, such as ischemic heart disease, valvular heart disease, hypertrophic cardiomyopathy, heart failure with preserved or moderately reduced LVEF, arterial hypertension, diabetes mellitus, cardiotoxic cancer therapy, in which LV-GLS has incremental prognostic role by reclassifying baseline function at every level of impaired left ventricular ejection fraction [74]. In this context, it would be reasonable to assume that LV-GLS could be a useful index for risk stratification on top of LVEF in β-TM patients in the near future. Finally, it should be mentioned that layer-specific strain evaluation using STE is an appealing concept due to the specificities of the cardiomyopathy of β-TM, as AbdelMassih et al. [32] have pointed out, despite the fact that its clinical availability is currently limited [75].

### Right ventricular strain

The number of studies evaluating RV-GLS is small and CMR was implemented only in half of these, thus no definite conclusions could be extracted. Until further studies are available, it could be considered that right ventricular deformation is being affected at a later stage compared to left ventricle [14]. This is attributed to the delayed myocardial deposition, the molecular differences in the isoforms of the heavy- chain of myoglobin and the consequent differences in shortening velocity and ATPase activity [76], [77], [78].

Regarding general population, there are significantly fewer studies on RV-GLS compared to LV-GLS and no clear reference values have been proposed due to high statistical heterogeneity between the studies [79]. The echocardiographic study and interpretation of the right ventricular mechanics require deep knowledge of the right ventricular pathophysiology that is multifactorial in nature [80]. Apart from the direct effect of iron toxicity, in advanced heart failure patients, right ventricular function could be affected either by precapillary (due to hemolysis) or post-capillary pulmonary hypertension (due to left ventricular systolic of diastolic dysfunction), thus further studies should include parameters such as hemolysis indices, invasive and non-invasive indices of left ventricular diastolic function and left ventricular systolic indices.

### Atrial strain

A few studies have evaluated left atrial deformation and there is heterogeneity in calculation methodology, as some authors have reported positive, negative and total left atrial strain, while others LASr, LAScd and LASct. It would be reasonable to assume that total atrial strain corresponds to LASr, positive strain corresponds to LAScd and negative strain to LASct. However, there is no evidence to support the validity of the aforementioned correlations, yet not overlooking the fact that LAScd has actually a negative value. Nevertheless, all case–control studies in β-TM concurred that patients have worse atrial deformation indices, apart from strain at atrial contraction phase. There are scarce data in literature on the effect of anemia on atrial strain. Shen et al. [81] have shown that hemoglobin concentration less than 9 g/dL was accompanied by decreased left atrial strain in patients with iron-deficiency anemia. In terms of myocardial iron load, LASr was found lower in half of the related studies. The findings from Cheung et al. [46] are suggestive of atrial cardiomyopathy in β-TM that is dissociated to ventricular mechanics [82]. However, left atrial deformation could be affected not only by primary atrial cardiomyopathy, but due to left ventricular dysfunction, as being reported by Karamanou et al. [52]. It should be taken into consideration that E/e’ index is not enough to determine the diastolic properties of the left ventricle among β-TM due to altered hemodynamics. Moreover, even the combined use of traditional echocardiographic indices (E/e’, septal e’, left atrial volume, pulmonary vein pulse wave doppler pattern) leaves a grey-zone in diagnosis of left ventricular diastolic dysfunction and LASr has been proposed as a novel index for clarification of these undetermined cases [83]. Future studies should implement a combination of these non-invasive echocardiographic indices or even invasive parameters (pulmonary capillary wedge pressure, left ventricular end-diastolic pressure) alongside left atrial strain to clarify the exact relation between atrial mechanics and diastolic function among β-TM patients.

Furthermore, Patsourakos et al. [54] reported worse left atrial mechanics among patients with prior episodes of atrial fibrillation compared to patients without. In a similar fashion, Vlachou et al. [53] associated left atrial strain to electrical atrial ectopy. There is evidence on heart failure with preserved ejection fraction that LASr provide incremental predictive information about incident atrial fibrillation [84]. These interesting findings suggest that β-TM patients with impaired LASr should be considered having altered atrial substrate that is arrhythmogenic and consequently should be prompted for further arrhythmiological evaluation. Finally, it should be noted that studies on pediatric β-TM patients regarding atrial mechanics are lacking.

### CMR imaging

Undoubtably, CMR T2* has a major impact on β-TM patient management. Nevertheless, CMR imaging is a very potent diagnostic tool and should not be limited only in myocardial iron load estimation. Multiple CMR techniques should be used to address specific issues, such as ECV estimation in patients with heart failure and no concurrent evidence of myocardial iron overload. These advanced CMR indices should be evaluated in order to accurate define the clinical impact in this specific population. Eventually, both 1.5 Tesla and 3.0 Tesla MRI scanners could be proposed in the near future to evaluate myocardial indices beyond T2*.

### Recommendations

It is suggested that LV-GLS being calculated either by 2D-STE from three apical echocardiographic views or by 3D-STE. Left ventricular strain should be evaluated for early detection of left ventricular dysfunction in β-TM patients, especially in patients younger than 18 years. Patients with impaired LV-GLS should be prompted to CMR imaging to estimate myocardial iron load and have a closer follow-up by a cardiologist.

Left atrial strain should be evaluated at reservoir phase and used as a screening tool to identify β-TM patients with increased electrical atrial ectopy and of increased possibility to develop atrial tachyarrhythmias (in particular atrial fibrillation). It is advised that LAS curve pattern is being assessed as well. These patients should be monitored with regular continuous electrocardiography for arrhythmia detection and initiation of appropriate medical treatment (antiarrhythmics, anticoagulants).

## Conclusion

Left ventricular deformation indices should be used to recognize early left ventricular dysfunction in β-TM patients. Further studies with larger patient groups should be designed to clarify the role of STE in detection of myocardial iron overload. Moreover, future studies on right ventricular strain should include hemolysis indices, as well invasive and non-invasive indices of left ventricular diastolic function. Finally, left atrial strain could be used not only as an additional left ventricular diastolic index, but as a screening tool to detect patients with high probability of developing supraventricular tachyarrhythmias as well.
